# Quantifying transmissibility of SARS-CoV-2 and impact of intervention within long-term healthcare facilities

**DOI:** 10.1098/rsos.211710

**Published:** 2022-01-12

**Authors:** Jessica E. Stockdale, Sean C. Anderson, Andrew M. Edwards, Sarafa A. Iyaniwura, Nicola Mulberry, Michael C. Otterstatter, Naveed Z. Janjua, Daniel Coombs, Caroline Colijn, Michael A. Irvine

**Affiliations:** ^1^ Department of Mathematics, Simon Fraser University, Burnaby, Canada; ^2^ Faculty of Health Sciences, Simon Fraser University, Burnaby, Canada; ^3^ Pacific Biological Station, Fisheries and Oceans Canada, Nanaimo, Canada; ^4^ Department of Biology, University of Victoria, Victoria, Canada; ^5^ Department of Mathematics and Institute of Applied Mathematics, University of British Columbia, Vancouver, Canada; ^6^ School of Population and Public Health, University of British Columbia, Vancouver, Canada; ^7^ British Columbia Centre for Disease Control, Vancouver, Canada; ^8^ British Columbia Children’s Hospital Research Institute, Vancouver, Canada

**Keywords:** COVID-19, reproduction number, outbreak modelling, transmissibility, long-term healthcare

## Abstract

Estimates of the basic reproduction number (*R*_0_) for COVID-19 are particularly variable in the context of transmission within locations such as long-term healthcare (LTHC) facilities. We sought to characterize the heterogeneity of *R*_0_ across known outbreaks within these facilities. We used a unique comprehensive dataset of all outbreaks that occurred within LTHC facilities in British Columbia, Canada as of 21 September 2020. We estimated *R*_0_ in 18 LTHC outbreaks with a novel Bayesian hierarchical dynamic model of susceptible, exposed, infected and recovered individuals, incorporating heterogeneity of *R*_0_ between facilities. We further compared these estimates to those obtained with standard methods that use the exponential growth rate and maximum likelihood. The total size of outbreaks varied dramatically, with range of attack rates 2%–86%. The Bayesian analysis provided an overall estimate of *R*_0_ = 2.51 (90% credible interval 0.47–9.0), with individual facility estimates ranging between 0.56 and 9.17. Uncertainty in these estimates was more constrained than standard methods, particularly for smaller outbreaks informed by the population-level model. We further estimated that intervention led to 61% (52%–69%) of all potential cases being averted within the LTHC facilities, or 75% (68%–79%) when using a model with multi-level intervention effect. Understanding of transmission risks and impact of intervention are essential in planning during the ongoing global pandemic, particularly in high-risk environments such as LTHC facilities.

## 1. Introduction

Early outbreaks of coronavirus disease 2019 (COVID-19) occurred in locations including public transit, places of worship, cruise ships, meat-packing plants, ski resorts, prisons and fishing vessels [1–7]. Prisons have also been highlighted as potential sources of high transmission due to housing an overcrowded ageing population with underlying health conditions [8]. Similarly, detention facilities have been noted as locations where rapid transmission could lead to hospitalizations exceeding local healthcare capacity [9]. Long-term healthcare (LTHC) facilities have also been sites of large COVID-19 outbreaks [10,11]. The virus has been observed to spread rapidly in the clustered susceptible population of LTHC facilities. Since most people in LTHC are old and frail, morbidity and mortality during an outbreak are often very high, with case fatality rates around 30% [12,13].

There has been considerable focus on estimation of the basic reproduction number *R*_0_ in different jurisdictions during the COVID-19 pandemic [3,14–18]. Given the high fatality rate, there is a keen interest in assessing the impact of COVID-19 on LTHC facilities [13,19–22]. However, to date there has been limited work on estimation of reproductive numbers or other transmission parameters within LTHC or other high-risk facilities. Estimates of transmission parameters are useful for retrospective analysis of the efficacy of interventions—particularly when preventative measures have changed between facilities or over time within one facility—and also in planning for future outbreaks. By comparing facility-specific estimates to those from the general population, we can also understand how transmission in LTHC environments differs.

Within British Columbia (BC), Canada, there have been 99 identified and defined ‘reportable’ outbreaks, including LTHC facilities, other acute care or assisted living facilities, workplaces, correctional facilities, and religious institutions as of 21 September 2020. Fifty-three of these outbreaks were in LTHC facilities; 18 of which included more than a single case of COVID-19, were complete by 21 September and were able to be linked to capacity data. Protocols for LTHC outbreak management have been established, including implementation of a suite of interventions once an outbreak has been identified within a facility [23,24]. These interventions include isolation of active cases, isolation of all residents in the facility, increased measures around infection control, reduced barriers to testing and increased testing frequency, and increased staffing and resources. Although the precise timing of the implementation of these interventions can vary, they typically occur one to two days after identification of a resident or staff case of COVID-19.

In this work, we estimate *R*_0_ in 18 BC LTHC COVID-19 outbreaks using a dynamic susceptible–exposed–infected–recovered (SEIR) model within a Bayesian hierarchical framework. A time-dependent infection rate in the SEIR model incorporates the implementation of COVID-19 interventions upon identification of each outbreak. Specifically, we estimate *R*_0_ for each outbreak in the period before full outbreak interventions were put in place, as well as the strength of such interventions. The hierarchical framework allows for each LTHC outbreak to be analysed within the context of all other outbreaks in the dataset, and is therefore useful for small outbreaks where the information available from each outbreak is limited. Use of a Bayesian model also allows for the incorporation of prior knowledge into the analysis, as well as the ability to fully explore the posterior distribution of the estimates. Previous modelling work has, for example, incorporated a chain binomial transmission model within a Bayesian hierarchical model in order to incorporate multiple households into one framework [25], or sought to estimate *R*_0_ within a Bayesian hierarchical framework using the final size of an outbreak rather than incorporating a disease dynamic model [26]. Disease dynamic models have also been used within Bayesian hierarchical frameworks for forecasting seasonal influenza [27]. In this work, we also compare the estimates from the Bayesian hierarchical model to those from several established statistical approaches for *R*_0_ estimation, which consider each LTHC outbreak independently. We lastly investigate if the heterogeneity in our estimates of *R*_0_ is explainable by factors such as the outbreak date or the facility capacity, or if this is likely a result of stochasticity in transmission.

## 2. Methods

### 2.1. Data

Data on reported outbreaks within BC were identified through the BC Centre for Disease Control. Those outbreaks that were identified as having taken place within a LTHC facility, that were able to be linked to facility capacity information, that were complete by the end of the study period, 21 September 2020, and that had more than one case were selected, for a total of 18 outbreaks. During the study period, no Variants of Concern had yet been identified in BC. These data consist of reported date, symptom onset date and facility location of all identified cases of COVID-19 (residents and staff). We also performed a supplementary analysis of the 30 identified single-case LTHC outbreaks meeting the above outbreak criteria, of which 25 were a staff member, 3 were residents and 2 were of an unrecorded source. These LTHC data were previously not publicly released; however, the de-identified data are now available as part of the Github repository attached to this work at github.com/sempwn/cr0eso [28].

Missing symptom onset dates (44/536 cases across all facilities) were replaced by selecting a symptom onset date uniformly at random from all known symptom onset dates within the same facility. This results in a higher chance of sampling more common symptom onset dates within each facility. Sensitivity analysis to this interpolation was performed, in which we re-sampled the missing data 100 times and explored the impact on the facility-specific *R*_0_ estimates. The start time of each outbreak was selected as the earliest symptom onset date within all identified cases in that outbreak. The reported date for each outbreak was recorded as the earliest reported date among all reported cases in the facility, since in BC one case in a LTHC facility is sufficient to be labelled as an outbreak.

Additional covariate data concerning the LTHC facilities included in this study were obtained from the Office of the Seniors Advocate, BC, website [29]. Where available, we compiled data on various factors for each facility: average resident age, average resident stay, direct care hours, facility capacity, number of recorded disease outbreaks, number of lodged complaints, proportion of residents dependent on assistance for their daily activities, age of facility, and accreditation status. These data are from 2018/19, the most recent year available. This was combined with two additional factors concerning the outbreaks: the outbreak reported date and identity of the initial COVID-19 symptomatic case (staff, resident or unknown).

### 2.2. Bayesian hierarchical model

We introduce a novel modelling framework for estimating *R*_0_ in LTHC facilities, by incorporating a dynamic compartmental SEIR model into a Bayesian hierarchical structure that includes both parameter and observation uncertainty. This framework incorporates data on each outbreak, allowing for a more robust approach to estimation than considering each outbreak separately, such that outbreaks with few cases can depend strongly on a prior distribution of *R*_0_. Our methodology combines the flexibility of dynamic compartmental models for transmission, allowing for estimation not just of the underlying transmission parameters but also the strength of interventions, with the benefits of Bayesian hierarchical fitting such as increased robustness of estimates and incorporation of prior knowledge.

The baseline transmissibility of COVID-19 in our model was assumed to vary between facilities due to differences in layout, contact structure, and demographics of the facilities. Within BC, standard policies seeking to reduce transmission were implemented across the province [23,30]. We therefore modelled the interventions as having equal strength in all facilities under the assumption that implementation occurred within one day of the first reported case. We also performed a sensitivity analysis in which the intervention strength was allowed to vary between facilities.

#### 2.2.1. Transmission model

We begin with a standard SEIR model where the transmission term in a facility is dependent on the time since identification of the first case in the facility. An individual in facility *k* starts out as susceptible to COVID-19 (*S*_*k*_), following an infection they transition to an exposed group (*E*_*k*_), after an incubation period the individual transitions to an infected group (*I*_*k*_), before finally transitioning to a recovered group; *S*_*k*_, *E*_*k*_ and *I*_*k*_ represent the number of individuals in each group. Due to the short nature of a facility outbreak, imported and exported cases were not considered. Each outbreak was considered to be initiated by a single infectious individual. During the period of this study, general transmission in the community was low, giving a small risk of further importation over a short period. We incorporated uncertainty into the facility population sizes in part to account for staff, but otherwise did not model staff–resident contact any differently from resident–resident contact.

The model equations can be represented as

$$\;\frac{dS_{k}}{dt}=-\frac{R_{k}(t)\gamma }{N_{k}}S_{k}I_{k},$$

$$\frac{dE_{k}}{dt}=\frac{R_{k}(t)\gamma }{N_{k}}S_{k}I_{k}-\sigma E_{k}$$

$$\;\mathrm{and}\;\;\frac{dI_{k}}{dt}=\sigma E_{k}-\gamma I_{k},$$

where *R*_*k*_(*t*) is the effective reproduction number in facility *k* at time *t*, *γ* is the recovery rate, *σ* is the incubation rate (the rate at which latent individuals become infectious) and *N*_*k*_ is the population size which is approximated by the capacity of facility *k*. After detection of the first case in a facility, interventions were implemented aiming to reduce the infectiousness within the outbreak cluster. We modelled this with an *R*_*k*_(*t*) term that incorporates the initial reproduction number *R*_0,*k*_ in facility *k*, and the modified reproduction given intervention. Assuming that outbreaks are eventually brought under control leading to *R*_*k*_(*t*) < 1, *R*_*k*_(*t*) can be defined using the form

$$R_{k}(t)=\left\{ \begin{array}{cc} R_{0,k} & t\leq \tau_{k}\;\\ R_{0,k}\;e^{\zeta (\tau_{k}-t)} & t>\tau_{k}, \end{array}\right.\;$$

where *ζ* is the intervention effect rate and *τ*_*k*_ is the time of the first case reported within the facility.

#### 2.2.2. Parameter model

The parameter model incorporates the above transmission model with a multi-level *R*_0_ term that is informed by data from all LTHC facilities in the study. More concretely, for outbreak *k*, an *R*_0,*k*_ is drawn from the distribution:

$$R_{0,k}∼N(R_{0},\sigma_{R}^{2})[0,\infty ],$$

where *R*_0_ is the population-level basic reproduction number before outbreak detection, and the standard deviation *σ*_*R*_ is drawn from a standard half-normal prior [31].

The literature-based priors were selected for the population-based reproduction number *R*_0_, the infectious period (recovery rate), and the incubation period. As these parameters represent an estimate of the population mean, their variances were characterized by the literature-based standard error, making them highly informative. The mean rate of incubation prior (*σ*) had a mean of 0.2 (corresponding to an incubation period of 5 days) and standard deviation 0.025 (providing a 95% CI of approx. 4.0–6.6 days) [32]. The mean rate of recovery (*γ*) had a prior mean of 0.125 (corresponding to an infectious period of 8 days) with a standard deviation of 0.0125 (providing a 95% CI of approx. 6.7–10.0 days). The prior for *R*_0_ was given a mean of 3.0 with a standard deviation of 1.0 to account for a population mean that may be higher than observed in general community transmission [33]. Other parameters, including the between-facility variance in *R*_0_, $\sigma_{R}^{2}$, and timing and strength of the intervention, had more uncertainty in their prior distributions. To capture uncertainty in the facility population sizes, the population size of facility *k*, *N*_*k*_, was given a normally distributed prior with mean equal to the known facility capacity ${\hat{N}}_{k}$ and standard deviation 10. The prior for the initial proportion of susceptible individuals *S*_*k*_(0) was selected such that the mean would be 99 individuals in a population of 100 with a standard deviation of one person (so the mean number of initially infective individuals is one in a population of 100).

The full Bayesian model (without the data likelihood, which is described below) is as follows; all rates have units day^−1^:

$$R_{0}∼N(3,1)[0,\infty ],$$

$$\sigma_{R}∼N(0,1)[0,\infty ],$$

$$R_{0,k}∼N(R_{0},\sigma_{R}^{2})[0,\infty ],$$

$$\gamma ∼N(0.125,0.0125^{2})[0,\infty ],$$

$$\sigma ∼N(0.2,0.025^{2})[0,\infty ],$$

$$N_{k}∼N({\hat{N}}_{k},10^{2})[0,\infty ],$$

$$S_{k}(0)∼N(0.99,0.01^{2})[0,\infty ],$$

$$\tau_{k}∼N(0.1,1)[0,\infty ]$$

$$\;\mathrm{and}\;\zeta ∼N(0.1,0.1^{2})[0,\infty ].$$

The likelihood is constructed by treating the modelled daily incidence for each facility as a Poisson-distributed random variable, with the rate given as the rate of transition from the *E*_*k*_ to *I*_*k*_ class ($\iota_{k}(t)$). Data on reported onset of symptoms on a given day for each facility (*C*_*k*_(*t*)) were used as opposed to date of test, as often tests were conducted en masse at a facility once a primary case had been identified. Although this does not explicitly model presymptomatic transmission, which has been found significant for COVID-19 [34], we incorporated additional variability in our incubation and infectious period priors to account for this. And indeed, if onset of infectiousness consistently lags onset of symptoms, our outbreaks would be shifted in time but otherwise unchanged. Cases were therefore sampled according to the following to construct the likelihood:

$$C_{k}(t)∼\mathrm{Poi}(\iota_{k}(t)).$$

Sampling of the posterior was performed with no U-turn sampling (NUTS) [35] using four chains, a warm-up period of 1000 samples, followed by 1000 iterations per chain. Visual inspection of the chains, pair-plots, Gelman–Rubin (split-chain-$\hat{R}$) statistic, and the presence of divergent transitions were used to assess convergence and mixing [36]. All Bayesian analysis was conducted using the Stan library [37], and our model is provided as an R package accompanying this article at github.com/sempwn/cr0eso [28].

As a sensitivity analysis to understand the potential heterogeneity of the effect of intervention, the model was extended to include a hierarchical structure for the intervention effect *ζ*. The hyper-priors were kept the same as the *ζ* prior in the original model, with inter-facility variance $\sigma_{\zeta }$ drawn from a standard half-normal prior.

Counterfactual scenarios were performed on all facilities by drawing a set of parameters from the posterior and then setting the intervention effect *ζ* to zero for the counterfactual parameter set. We numerically solved the SEIR model for each of the 4000 parameter sets and sampled from the model to produce a difference in total incidence between the two scenarios.

Validation of the hierarchical Bayesian procedure was also performed using simulated data. We simulated 30 outbreaks from an SEIR model, each with *R*_0,*k*_ drawn from a N(3,9) distribution truncated at 0, Poisson-drawn incidence, and a fixed intervention implementation. Each *R*_0,*k*_ was then estimated within the Bayesian hierarchical framework described above.

### 2.3. Comparison with established *R*_0_ estimation methods

Established methods for estimating *R*_0_ include calculation from the attack rate, which is the total proportion of a fixed population that contracted the disease during an outbreak [38]. Here, we estimated the attack rate as the total number of reported cases in the outbreak divided by the maximum capacity of the facility. The relationship between attack rate *A* and *R*_0_, under the model of the general deterministic epidemic, is given through the transcendental equation [39,40]

2.1 $$\log(1-A)=-R_{0}A.$$

We estimated *R*_0,*k*_ for each facility from the attack rate using equation (2.1) with *A* = *A*_*k*_ and *R*_0_ = *R*_0,*k*_. As a sensitivity analysis, the number of exposed individuals (the denominator in the attack rate) was varied between 85% and 115% of the maximum capacity for each facility. However, this approach does not take into account that interventions were put in place during the outbreak, and so resulting estimates are not true basic reproduction numbers but instead represent an averaging across the entire outbreak.

The basic reproduction number in each outbreak was estimated using two other approaches which consider the early growth period of an outbreak only: from the exponential growth (EG) rate [41] and from maximum likelihood (ML) [42]. These methods require an assumed generation time, for which a Gamma distribution with mean 5.2 and standard deviation 1.73, as estimated for COVID-19 in [43], was used. The EG method calculates the exponential growth rate *r*_*k*_ from the initial exponential period of outbreak *k*, which we define as the time from symptom onset of the first case to the maximum incidence day (if multiple days have maximum incidence, we pick the latest). Poisson regression is used to estimate *r*_*k*_, to account for the fact that the incidence data are integer valued. *R*_0,*k*_ is then calculated as 1/*M*(−*r*_*k*_), where *M* is the moment generating function of the generation time distribution. The ML method assumes that the offspring distribution is Poisson distributed with expectation *R*_0,*k*_. *R*_0,*k*_ is estimated by maximizing the resulting Poisson likelihood, given daily incident case counts. This estimation is also performed over an initial exponential period only, which we define as in the exponential growth method. Both the EG and ML approaches were implemented using the R0 library in R [44].

We performed an additional analysis using the number of single-case outbreaks (which could not be incorporated into the hierarchical model, EG or ML analyses due to their small size) to obtain a single *R*_0_ estimate for BC LTHC outbreaks in the study period, by relating *R*_0_ to the observed number of outbreaks which did not lead to any within-facility transmission. Details of this analysis are provided in electronic supplementary material, section S1.

### 2.4. Correlations with outbreak characteristics

We calculated Pearson correlation coefficients (PCCs) and corresponding 95% confidence intervals between the facility-specific *R*_0,*k*_ values estimated from the hierarchical model and various other covariate data concerning the LTHC facilities as listed in §2.1.

All analyses were run using R v. 4.0.0 [45,46].

## 3. Results

**Figure 1.**
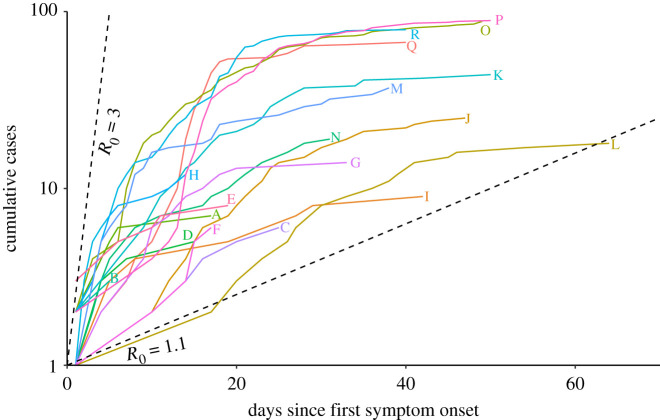
Cumulative cases from 18 LTHC outbreaks in BC, Canada. Cumulative number of cases for each identified outbreak is plotted against symptom onset. Early growth of cases will follow a straight line on the semi-log scale if growth is exponential. Cumulative cases are displayed as linearly increasing between successive symptom onset dates within the facility to aid in this visualization. Dashed lines represent unimpeded epidemics with *R*_0_ values of 1.1 and 3, with a serial interval of 5 days.

**Figure 2.**
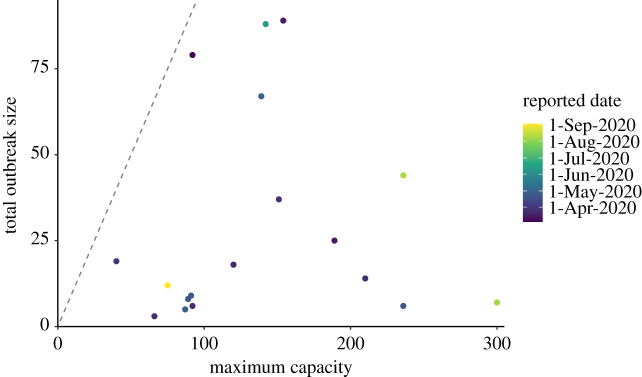
Total outbreak size by facility capacity of LTHC facilities in BC, Canada. Total outbreak sizes of the 18 LTHC facilities are shown against the reported maximum capacity of that facility. Dashed line is the one-to-one line. We find no clear relationship between outbreak size and capacity or reported date.

Data on 99 outbreaks within BC, Canada, as of 21 September 2020 were identified. Of these identified outbreaks, 53 were selected as having taken place within a LTHC facility. Of these, 4 were excluded as they had ambiguous names or were otherwise not able to be linked to the facility characteristics data, 30 were excluded as only having one case, and 1 was excluded as still ongoing, resulting in 18 identified LTHC outbreaks. Facilities were anonymously labelled alphabetically by increasing mean *R*_0,*k*_ from the Bayesian hierarchical model. Outbreak duration and size varied greatly between locations with a duration of outbreak between 10 and 64 days (figure 1) and size of outbreak between 3 and 89 cases (figure 2; electronic supplementary material, table S2), leading to an overall attack rate between 2% and 86% (electronic supplementary material, table S2). Outbreak size variation showed no clear pattern by reported facility size (figure 2).

**Figure 3.**
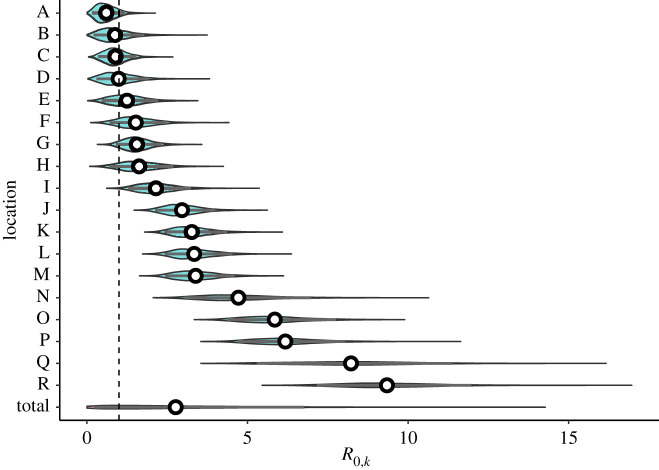
Hierarchical model fitting of *R*_0,*k*_. Each outbreak in an LTHC facility where the outbreak was greater than one individual was incorporated into the Bayesian hierarchical SEIR model that accounted for increased control measures once the first case had been identified. The posterior distributions of the *R*_0,*k*_ estimates from 4000 samples are shown, with the posterior for *R*_0_ (total) shown below. Median values are shown as white points, with the 90% credible interval as grey bars, and the general distributions shown as density violin plots. Vertical dashed line is *R*_0,*k*_ = 1.

**Figure 4.**
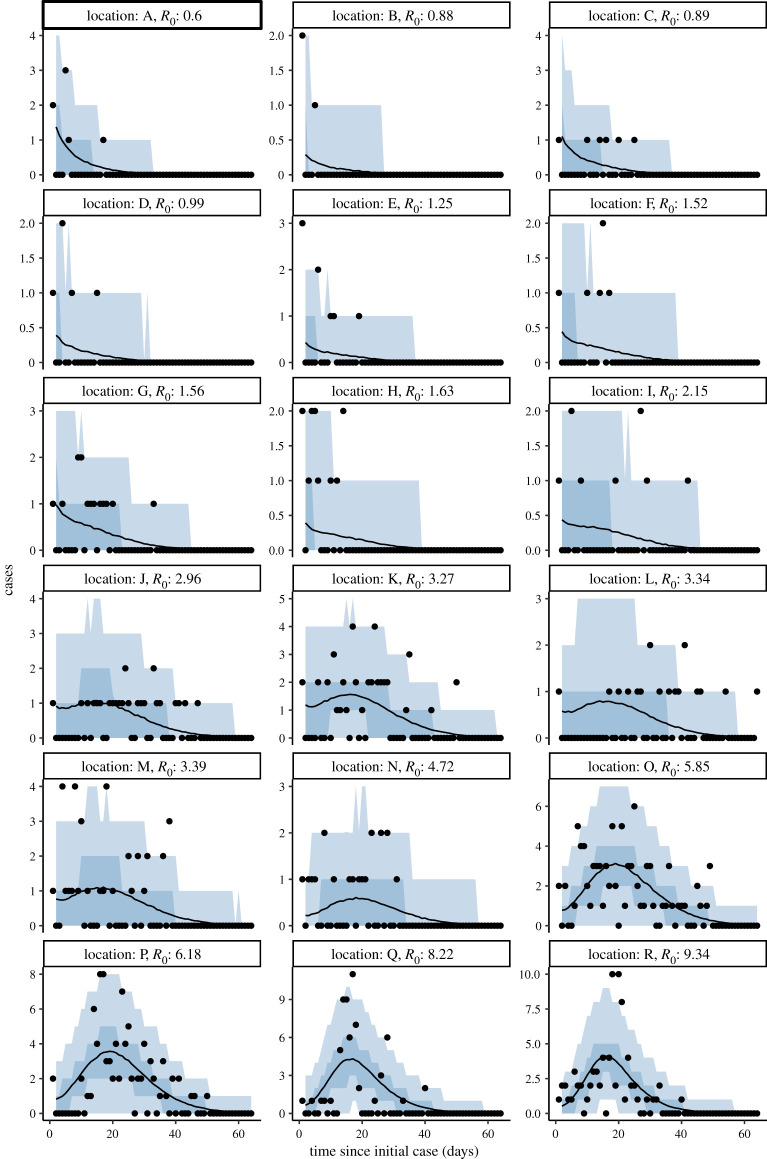
Bayesian hierarchical model fits to incident cases. For each LTHC outbreak of size greater than one, the posterior predictive distribution from 4000 samples is shown. Median posterior values are shown as lines with the 50% and 90% credible intervals shown as darker and lighter shaded regions, respectively. Observed cases are overlaid as black points. Facilities are ordered by increasing mean *R*_0,*k*_.

Bayesian hierarchical model fitting of *R*_0_ found a large range of estimates by location, with 4 of 18 locations having median *R*_0,*k*_ below the critical threshold of 1, but all of these having *R*_0,*k*_ = 1 within the 90% credible interval (figures 3 and 4; electronic supplementary material, table S2). The overall predictive *R*_0_ mean estimate was 2.51 (90% CrI (credible interval) 0.47–9.0), and the range of *R*_0,*k*_ estimates by location varied largely within this interval: from the lowest (location A) 0.56 (0.16–1.17) to the highest (location R) 9.17 (7.16–11.97). The incorporation of a hierarchical structure for the intervention strength (*ζ*) increased the overall estimate of *R*_0_ to 4.07 (1.76–10.06) (electronic supplementary material, figures S1, S2 and table S2). The posterior predictive distributions of the LTHC outbreaks revealed tighter fits for larger outbreaks and more uncertainty for smaller ones, as would be expected (figure 4; electronic supplementary material, figure S2). Conversely, we did not find such uncertainty in the *R*_0,*k*_ estimates for smaller outbreaks (figure 3). This highlights a strength of this hierarchical approach: in using data from larger outbreaks to regularize fitting in smaller ones, despite larger levels of uncertainty. In model validation, applying our approach to 30 simulated outbreaks, the hierarchical modelling procedure was able to recover the data-generating parameters (electronic supplementary material, figure S3).

**Figure 5.**
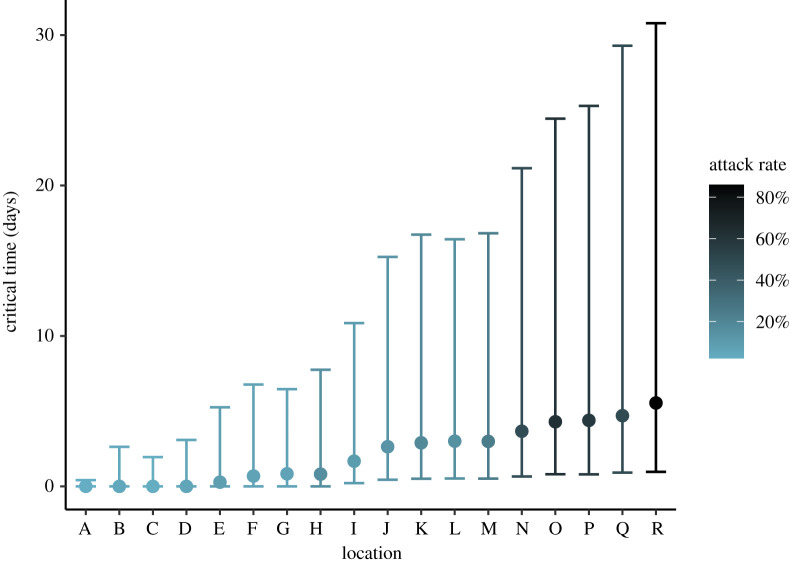
Posterior median estimates of the critical time from outbreak reported date to the reproduction number falling below 1. Calculated from 4000 posterior draws of *R*_0,*k*_ and *ζ*, with error bars showing 90% credible intervals.

Using the Bayesian hierarchical model, we also estimated the ‘critical time’ in each facility. That is, we took samples from the posterior of the length of time it took for the reproduction number to fall from *R*_0,*k*_ to below 1, once each outbreak was reported and full-scale interventions were implemented. The critical time varied with *R*_0,*k*_, but ranged between 0.0 days (for outbreaks with *R*_0,*k*_ already below 1) and 5.54 days (figure 5; electronic supplementary material, table S2). There was considerable uncertainty in these estimates, however. Counterfactual modelling was conducted to estimate the impact of intervention on the total number of cases averted compared to an unhindered outbreak. Across all outbreaks, 61% (90% CrI 52%–69%) of all potential cases were estimated to have been averted (electronic supplementary material, figure S4). This corresponds to 890 cases (90% CrI 658.95–1184.05) avoided across the 18 outbreaks. Within each facility the impact of intervention varied: the highest at location I with 84% (90% CrI 67%–93%) cases averted and the lowest at location R with 9% (90% CrI −18% to 31%) cases averted (electronic supplementary material, figure S5). Under the model with multi-level intervention *ζ*, counterfactual modelling suggested a larger proportion of potential cases were averted: 75% (68%–79%) across all outbreaks, corresponding to 1681 cases (90% CrI 1215–1909), and mostly a result of a higher estimated proportion in the smallest outbreaks A–H (electronic supplementary material, figures S6 and S7).

**Figure 6.**
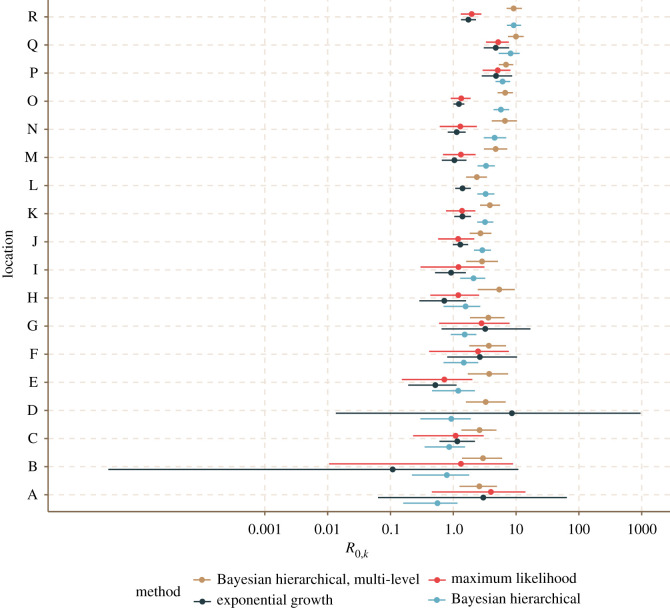
Comparison of *R*_0,*k*_ estimates for COVID-19 outbreaks in LTHC facilities in BC, Canada, ordered from top to bottom as follows: Bayesian hierarchical multi-level median, maximum likelihood, exponential growth, Bayesian hierarchical median. Note the log-distributed axis. For each facility, *R*_0,*k*_ estimates with 95% confidence interval (exponential growth and maximum likelihood) or 90% credible interval (Bayesian hierarchical models, with and without multi-level *ζ* term, 4000 samples) are shown.

The *R*_0,*k*_ estimates obtained from the EG and ML methods were quite different from those from the hierarchical model for many facilities (figure 6; electronic supplementary material, table S2), highlighting issues with fitting these (often small) outbreaks independently. Several of the smaller outbreaks (facilities A, B, D, F, G) had a large degree of uncertainty around the EG and ML estimates, which was not seen in the regularized estimates from the hierarchical model. For two outbreaks (facilities D and L) the ML method was unable to converge on an estimate: potentially due to the very fast (facility D) or slow (facility L) take-off of cases between the start of the outbreak and the maximum incidence day which we defined as the end of the exponential growth period used in fitting. The hierarchical models did not suffer from such problems. There was limited concurrence between different methods as to the outbreaks with subcritical (less than 1) *R*_0,*k*_ (EG: B, E, H, I; ML: E; Bayesian hierarchical model: A, B, C, D). We see a general trend that for those outbreaks in which the hierarchical model estimated a smaller *R*_0,*k*_, the EG and ML methods, unguided by any prior or hierarchical information, have large levels of uncertainty (figure 6, ordered by increasing hierarchical model *R*_0,*k*_). For locations with larger *R*_0,*k*_ from the hierarchical model, we see consistently lower estimates from the EG and ML methods. This may be a consequence of the EG and ML methods working with the initial exponential period of each outbreak rather than incorporating a specific mechanism to model that interventions were put in place, unlike the hierarchical models. The impact of this may be more pronounced in those longer and larger outbreaks with higher *R*_0_.

To explore the impact of interpolating the missing symptom onset dates on the EG and ML *R*_0,*k*_ estimates, we used a Monte Carlo procedure for repeated random sampling of the missing dates. The resulting Monte Carlo means and confidence intervals can be found in electronic supplementary material, table S1. We find that, despite the wide confidence intervals for some outbreaks in electronic supplementary material, table S2, the results are fairly robust to different interpolations of the missing symptom onset times (and many outbreaks have no missing symptom onset times). This would suggest that the majority of the uncertainty in the *R*_0,*k*_ estimates does not stem from the missing data, but from some other limitation(s) of the methods.

We calculated an additional population-level estimate of *R*_0_ from the number of outbreaks which had only one case, i.e. those in which no within-facility transmission was observed. Full results are provided in the electronic supplementary material, section S1. We found that if all 30 identified single-case outbreaks had a fully infectious individual present in the facility, the corresponding estimate of *R*_0_ is 0.6 (95% CI 0.33–1.08). This is at odds with the large number of cases observed in many of the facility outbreaks, and suggests (a) large heterogeneity in transmission of SARS-CoV-2, as has been been identified in other studies [47,48] and (b) many of the identified single cases may not have been fully infectious inside the facility. In fact, we note that 25 of the single-case outbreaks were in staff members (3 were in residents, and 2 were not recorded). It is not known whether these staff members attended the facility during their infectious period, or if an outbreak was declared out of an abundance of caution. *R*_0_ = 0.6 (95% CI 0.33–1.08) therefore represents a minimum estimate from these single-case data, with the maximum estimate being *R*_0_ = 6.0 (95% CI 2.0–22.8) corresponding to only the three resident cases being infectious within the facility. There is clearly considerable uncertainty here, which cannot be refined without additional information on the single-case outbreaks.

We include estimates of *R*_0,*k*_ directly from the attack rate in electronic supplementary material, table S2, though it is important to note that these do not truly represent ‘*R*_0_’ since they do not take into account that interventions were implemented and consequently represent an averaging across the whole outbreak. Nonetheless, in the absence of temporal data this may be the only way to estimate *R*_0_ and so we include these estimates for reference. Although we would therefore not expect *R*_0_ estimates from the attack rate to be directly comparable to those from the other methods, we did find significant correlation (PCC 0.94) between the *R*_0,*k*_ estimates from the Bayesian hierarchical modelling and the inferred attack rates (electronic supplementary material, figure S8), which is not found between the EG and ML *R*_0,*k*_ estimates and the attack rate (PCC 0.04 and 0.27). This is a result in part of the varying and high-uncertainty estimates of *R*_0_ for smaller outbreaks under the EG and ML approaches.

We found only limited correlation between our estimates of *R*_0,*k*_ from the Bayesian hierarchical model and other factors regarding the LTHC facilities and their COVID-19 outbreaks, obtained from the Office of the Seniors Advocate, BC [29] (electronic supplementary material, tables S3 and S4, figure S9). No significant correlation was found between *R*_0,*k*_ and the initial case in a facility (by symptom onset date) being a staff member (PCC 0.15). Though, as we do not model healthcare workers any differently from LTHC residents, our model framework is not ideal for this comparison. For the continuous variables tested, all 95% confidence intervals on the correlation coefficient included zero, with the exception of the number of lodged complaints during year 2018/19 for which we found a statistically significant negative correlation with *R*_0,*k*_ (PCC −0.54, *p*-value 0.04). Other small to moderate correlations were found between *R*_0,*k*_ and the average stay of residents in the LTHC facility (PCC 0.38), the number of direct care hours allocated per resident per day (PCC 0.4) and, to some extent, a negative correlation between *R*_0,*k*_ and the date on which the COVID-19 outbreak was reported in the facility (PCC −0.25), suggesting that later outbreaks were associated with a lower *R*_0,*k*_ despite the uniform intervention policy in place in all facilities throughout the study period. However, *p*-values for these three tests were all above 0.05, and the sample size is relatively small; furthermore, we were unable to obtain complete data on all facilities.

## 4. Discussion

In this paper, we characterized variation in the estimated *R*_0_ within a series of COVID-19 outbreaks in LTHC facilities. As *R*_0_ is a product of infectiousness of the pathogen, contact rate and type of contact, it is important to characterize *R*_0_ in order to understand the range of potential scenarios and total cases that could occur in future outbreaks, as well as subsequent capacity needs for healthcare provision, hospitals and intensive care units. Our Bayesian hierarchical model was able to regularize model fits even for outbreaks with a small number of cases, compared to the non-hierarchical methods for *R*_0_ estimation which were independent between facilities and consequently suffered from high levels of uncertainty. This highlights a need for methods that consider totality of evidence. Our estimates of *R*_0_ are specific to BC LTHC, and for many reasons could be expected to differ from estimates in the wider population. Nonetheless, the overall estimate of *R*_0_ = 2.51 (0.47–9.0) agrees relatively well with published studies [15], albeit with wide uncertainty induced in part by widely varying individual facility estimates. In addition to strong performance, demonstrated by the model validation results, this is a flexible framework that could be adapted for different types of outbreak outside of LTHC such as schools.

We found pronounced variability in *R*_0_ estimates by LTHC facility, despite all outbreaks occurring in facilities with the same provincial standards and with wild-type SARS-CoV-2. Variation in *R*_0_ estimates could not be explained by facility size or whether the first infected individual was a healthcare worker. The only statistically significant relationship found was a negative correlation between *R*_0_ and the number of complaints lodged against the facility (PCC −0.54). Further investigation would be required to explain this, but nonetheless, the majority of the variation in *R*_0_ estimates by facility was not found to be explainable by the factors considered, nor by the missing data imputation as explored in the sensitivity analysis. Given the absence of explainable factors, this supports previous findings of the presence of super-spreader events and high natural heterogeneity between outbreaks of COVID-19 [47,48], which should be taken into account when modelling. Particularly in small outbreaks, stochastic effects can dominate, where random events can lead to a large number of secondary cases. It is important to note that our estimates do not explicitly model such events, but rather estimate the total secondary cases in aggregate. Then, the variation in estimated *R*_0_ can be seen as explained by variation in these early events and in between-person transmissibility.

A counterfactual analysis of the scenario in which no interventions were put in place after the identification of an outbreak in each facility estimated that intervention led to 61% (52%–69%) of all potential cases being averted within LTHC facilities in BC (electronic supplementary material, figure S4). This increased to 75% (68%–79%) in the model with hierarchical intervention *ζ*. This suggests that fast intervention after outbreak identification is key to controlling the spread of COVID-19. Estimates of the ‘critical time’ between implementation of interventions and the reproduction number dropping below one were all less than 6 days; however, the credible intervals were often large, up to around 30 days in the outbreaks with highest attack rate. Particularly given the high fatality rates seen in LTHC facilities, implementing strong interventions rapidly will be particularly impactful in minimizing morbidity and mortality. Although the recommended suite of interventions remained constant throughout the study period, facility outbreaks from later in the study period were weakly associated with lower critical times (PCC −0.25), suggesting some improvement in the implementation of control measures over time.

There are limitations to the study. The reported attack rate and modelled population size for LTHC facilities were based upon the maximum capacity for that facility, which may be larger than the total number of exposed individuals. Conversely, staff and family members may contribute to transmission in addition to facility residents. Although we allowed the maximum capacity size to vary to account for these factors, we lacked detailed data to inform this. Similarly, we included staff cases in our analysis but did not consider a distinction between the behaviour of staff and residents. An improved model could take this, or other factors such as presymptomatic transmission for example, into account. However, this would need to be informed by additional data. Although we estimated parameter *ζ*, the rate at which interventions brought *R*(*t*) below 1, the manner in which intervention curtailed transmission was not explicitly explored within this study, because the exact nature of interventions and their timings were not precisely known. In our model framework, it is possible for *ζ* to be impacted by other factors, for example a saturation effect from a lack of available susceptible LTHC residents, rather than by intervention measures alone. However, this seems unlikely in this study, given the observed attack rates.

We considered all outbreaks as originating from a single index case in a closed population. Community prevalence remained low in BC during the period of this study so this seems a fair assumption, but it is possible that some facilities had multiple introductions. Asymptomatic cases were also not explicitly modelled in this work. It is not known if the missing symptom onset dates were caused by asymptomatic infections or data collection error, but given the low testing threshold and discretionary asymptomatic testing advised in BC LTHC facilities [49], it seems feasible that some asymptomatic cases were included in this dataset and therefore modelled the same as symptomatic cases. If many asymptomatic cases were missed however, this could be impactful on the estimates of the reproduction number. We removed all outbreaks of size one since a single data point does not contain enough information to fit the compartmental model. The additional analysis of these single-case outbreaks was not particularly informative without knowledge of how much opportunity for transmission these single cases had, exacerbated by 25/30 single case outbreaks being in staff members who may not have attended the facility while infectious. The assumption of homogeneity between outbreaks required for these calculations is also at odds with the motivations behind, and findings of, our main analysis, which suggested significant heterogeneity between locations.

The estimates of *R*_0_ in this work (from the initial period of each outbreak) do not represent the overall level of infectivity in the outbreaks, since interventions were swiftly implemented after the initial case was discovered. The attack rate was also low in many outbreaks, leading to potentially large effects of natural stochasticity on transmission. If trying to understand the potential for future outbreaks in LTHC, it is important to consider both *R*_0_ and *ζ* together. Importantly, we found that variation in *R*_0_ was not explainable by facility size or by the initial case being a healthcare worker, but limited data meant we were also limited in what covariates could be considered. Compared to some other jurisdictions, for example many areas of the USA [10], BC has seen a relatively small number of large outbreaks. With sufficiently detailed data, this analysis could be strengthened by considering other geographical locations. Other studies have considered overdispersion of secondary cases among outbreaks, finding high levels of overdispersion among country-level outbreaks more generally [50]. It is not clear currently how much of this heterogeneity in secondary cases is due to contact patterns, age of cases, or other biological factors. As in this study, we focused only on identified outbreaks and only on LTHC facilities, population-level overdispersion caused by sampling inconsistencies and differences in individual behaviour should be minimized, compared to country-level estimates for example. A negative binomial observational model could better incorporate remaining overdispersion caused by natural individual heterogeneity, but may lead to fitting challenges compared to the more restrictive Poisson model, particularly for smaller outbreaks as explored here. Overall, the Poisson model was found to provide a reasonable fit to the data.

For future LTHC outbreaks, measures such as regular testing of staff could assist with identifying potential outbreaks as early as possible. Across the 18 LTHC outbreaks in this study, two-thirds of those with known origin had a staff member as the initial case by symptom onset. At least 83% of those outbreaks with only 1 case had a staff member as the initial case. The analysis we have performed here could be refined with more accurate information on the total number of residents/staff per facility, as well as on differing levels of interaction between staff and residents. But also, more generally, prospective collection of data concerning factors that have been shown to impact spread of COVID-19 in other settings could help reveal sources of variation in transmissibility between different LTHC settings: including ventilation systems, staff deployment practices and patient layouts.

Despite evidence of improvement in the implementation of control measures in LTHC facilities over time, by the end of 2020 the number of reported long-term care facility outbreaks in BC increased significantly to 224 [51]. This may largely be due to increased community prevalence [51], increasing the rate of importation to LTHC facilities, but also highlights significant remaining challenges in outbreak management. Modelling an increased community prevalence of COVID-19 would require a generalization of our current approach, which does not consider repeat importations to each LTHC facility, by estimation of a rate of case importation. This is a topic for future study, but could be highly informed by the current model in which we can more reasonably assume a single point of introduction. The increase in both community prevalence and the number of facility outbreaks in BC also highlights that LTHC facilities are strongly connected to their surrounding community, and this community plays a large role in protecting LTHC facilities from outbreaks, of COVID-19 and beyond. Although our findings suggest that the suite of interventions implemented in BC LTHC facilities has been effective in significantly reducing the size of COVID-19 outbreaks, the high variability in the *R*_0_ estimates, despite uniform controls applied and lack of correlation to other available factors, suggests that early events (before outbreak detection) and large heterogeneity in between-person transmissibility are highly impactful. With regions including BC still facing significant COVID-19 case counts despite vaccine uptake [52], combined with concerns over waning vaccine-induced immunity and weaker immune response in the elderly [53], our findings suggest that further improving controls, particularly before outbreaks begin, is key to reducing disease burden and associated mortality in LTHC facilities under increased community transmission.

## Data accessibility

Data and relevant code for this research work are stored in GitHub: github.com/sempwn/cr0eso and have been archived within the Zenodo repository: https://doi.org/10.5281/zenodo.5722322 [28]. Covariate LTHC facility data were obtained from https://www.seniorsadvocatebc.ca/quickfacts/location [29]. Further cumulative data on LTHC outbreaks in BC are available from http://www.bccdc.ca/health-info/diseases-conditions/covid-19/data [52].
